# Electron Tomography Reveals Novel Microtubule Lattice and Microtubule Organizing Centre Defects in +TIP Mutants

**DOI:** 10.1371/journal.pone.0061698

**Published:** 2013-04-16

**Authors:** Johanna L. Höög, Stephen M. Huisman, Damian Brunner, Claude Antony

**Affiliations:** 1 Sir William Dunn School of Pathology, University of Oxford, Oxford, United Kingdom; 2 Laboratory for 3D Structure of Cells and Molecules, Department of Molecular, Cellular, and Developmental Biology, University of Colorado, Boulder, Colorado, United States of America; 3 Cell Biology and Biophysics, European Molecular Biology Laboratory, Heidelberg, Germany; 4 Institute of Molecular Life Sciences, University of Zürich, Switzerland; Université de Nice-CNRS, France

## Abstract

Mal3p and Tip1p are the fission yeast (*Schizosaccharomyces pombe*) homologues of EB1 and CLIP-170, two conserved microtubule plus end tracking proteins (+TIPs). These proteins are crucial regulators of microtubule dynamics. Using electron tomography, we carried out a high-resolution analysis of the phenotypes caused by *mal3* and *tip1* deletions. We describe the 3-dimensional microtubule organization, quantify microtubule end structures and uncover novel defects of the microtubule lattices. We also reveal unexpected structural modifications of the spindle pole bodies (SPBs), the yeast microtubule organizing centers. In both mutants we observe an increased SPB volume and a reduced number of MT/SPB attachments. The discovered defects alter previous interpretations of the mutant phenotypes and provide new insights into the molecular functions of the two protein families.

## Introduction

Microtubule (MT) dynamic instability is regulated by a wealth of MT associated proteins (MAPs). A subgroup of MAPs has been found to localize to MT plus ends and are commonly called +TIPs [1]. EB1 has been described as the master controller of the +TIPs, recruiting other proteins such as CLIP-170 to the MT plus end [2]–[5].

EB-class proteins preferentially bind to MT plus ends directly, by interacting with GTP-tubulin [6], but also bind along the MT seam [1], [7]. The MT seam is the position along the tube were neighboring protofilaments align in a different orientation, A lattice, than along the rest of the tube, which consists of B lattice [8]. It has been suggested that EB1 binding at the A lattice may stabilize this potential weak spot in MTs [7].

In fission yeast, the EB1 homologue Mal3p and the CLIP-170 homologue Tip1p have been shown to effectively stabilize MTs *in vivo*, since MT bundles have a reduced length in *mal3Δ* and *tip1Δ* mutants [3], [9], [10]. However, there is evidence that Mal3p does not stabilize MTs per se but rather inhibits shrinkage and promotes rescue along the tube, presumably via its lattice binding properties [11]. In addition, Mal3p also promotes MT nucleation *in vitro* [12].

As well as decorating MT plus ends and the MT lattice [7], [12]–[14], EB1 family members have been shown to localize to MT minus ends *in vitro* [2], and to microtubule organizing centers (MTOCs) such as the spindle pole body (SPB) in yeasts and the centrosome in higher eukaryotes [14]–[19]. CLIP-170 homologues, in turn, also localize to kinetochores [20], and MTOCs [21]–[23].

At the MTOCs, +TIPs have important functions. For instance, the budding yeast (*S. cerevisiae*) CLIP-170 and EB1 homologues, Bik1p and Bim1p, are necessary for SPB binding and subsequent transport of spindle orientation factors to astral MT plus ends [18], [24], [25]. Furthermore, CLIP-170 has been shown to inhibit centrosome duplication in human cells [23]. Finally, EB1 anchors MT minus ends around mammalian centrosomes [15], [19], [26]. Together, these reports show roles of EB1 and CLIP-170 family members in spindle alignment, centrosome duplication, and microtubule organization at MTOCs.

However, many questions remain about the roles of +TIPs in MT anchoring and nucleation. Are EB1 and CLIP-170 structural MTOC components, a part of the peri-centriolar material surrounding MTOCs and/or bound to MT minus ends? Is the anchoring function, direct or indirect, caused by their regulatory functions on the MT network? In this study, we use a combination of light microscopy, electron microscopy and electron tomography, to reveal that the absence of the *S. pombe* +TIPs Mal3p and Tip1p causes structural alterations in the SPB of fission yeast, indicating that Mal3p and Tip1p are also structural SPB components. Tip1p seems important for MT nucleation, as bundles contain only half the number of filaments. Finally, MTs in both +TIP mutants show unexpected lattice defects, such as kinks and thinner than usual tubes, further expanding their roles as MT regulators.

## Results

### Electron Microscopy and Tomography Show Altered SPB Morphology in *mal3Δ* and *tip1Δ* Mutants

**Figure 1:**
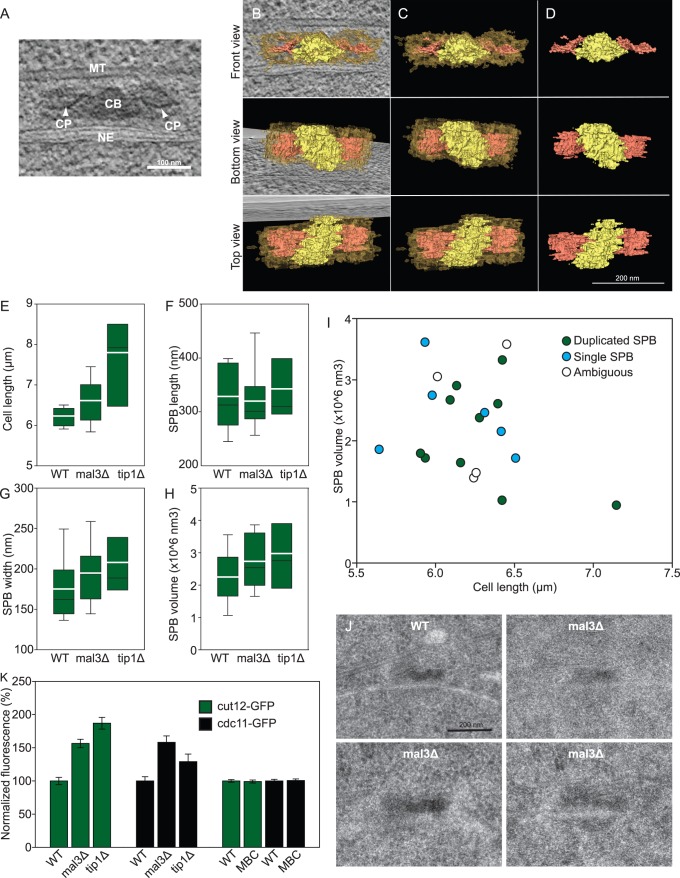
mal3Δ cells show altered SPB morphology and size. **A)** 1 nm thick section from a tomogram of a duplicated SPB. Central bridge (CB), central plaques (CP), microtubule (MT), nuclear envelope (NE). **B)** A 3D model of the duplicated WT SPB shown in A), amorphous SPB electron density represented in transparent gold, central bridge in yellow and the central plaques in red. The model is displayed with a slice from the electron tomogram, in which the NE and an associated MT shows in the front view. **C)** The same 3D model without the tomographic slice. **D)** The 3D model of the oblique central bridge and the central plaques only. **E)** The lengths of cells in which the SPBs were examined. **F–H)** All these measurements come from 3D models (made in IMOD) from serial thin section reconstructions of entire SPBs. Graphs show a comparison of the length, length and volume of the SPB 3D models. The black line across the boxes is the average, the white line the median, the box show the 25^th^ to the 75^th^ percentile. Error bars show the 5^th^ and 95^th^ percentile. **I)** A mixture of single and duplicated WT SPBs was found in early G2 cells, and their volume were measured and plotted against the cell length. No increase in volume is seen as the SPB duplicates. **J)** Thin section electron micrograph of WT and *mal3Δ* SPBs. The *mal3Δ* SPBs show abnormal morphology with unclear central plaques. **K)** Normalized fluorescence of the +TIP mutants and MBC treated cells compared to WT cells expressing the same fluorescent marker. The graph shows an increase in fluorescence for *mal3Δ* and *tip1Δ* mutants, not found in MBC treated cells. Error bars denote SEM, 90 or more cells/strain were analyzed.

In interphase *S. pombe* cells, SPBs are electron dense structures closely fitted between MTs and a mitochondrion on one side and the nuclear envelope on the other side [27], [28]; Figure 1A ). The amorphous electron dense material in the SPB is divided by an even more electron dense plaque, and an oblique central bridge, which connects the duplicated SPBs. However, the detailed 3D architecture of the *S. pombe* SPB has not been described. Here we show a reconstruction of a duplicated SPB, which reveals that the central bridge is an oblique structure wider than the flanking SPBs (Figure 1B–D; top and bottom views; Movie S1). The two central plaques are circular discs extending from the two upper ends of the central bridge. Each disc is slightly curved, concave to the nuclear envelope (Figure 1B–D; front view).

To scrutinize the *mal3Δ* SPB morphology closer, we examined serial-sections of 20 WT and 16 *mal3Δ* SPBs in synchronized early G2 cells (Figure 1E–J). The serial sections were used to create 3D reconstructions that were modeled and the dimensions of the SPBs were extracted from these models. The WT SPBs we reconstructed were between 233–495 nm long and 108–275 nm wide (average values were 328±67 nm long; 175±42 nm wide; Figure 1F–G). One SPB from a septating cell had an unduplicated SPB (Figure S1 in File S1) as expected. From cells in early G2 26% had not yet started to duplicate their SPBs. 53% of the SPBs had a clear secondary SPB ‘bud’ on the opposite side of the central bridge and 21% were not clearly identifiable as either single or duplicated SPBs. Note that the SPB volume is not directly correlated to its duplication state, indicating that the SPB grows by first initiating a bud, and then increases in volume (Figure 1I).

Electron micrographs show that SPBs in *mal3Δ* cells were more difficult to detect than in WT cells, the electron dense material appeared fluffy and the central plaques were often not visible (Figure 1J). SPBs in *mal3Δ* mutants were of similar length (320±58 nm) but slightly wider than WT SPBs (195±41 nm), they also appeared less electron dense, indicating a loosened protein structure. The total volume (2.73±0.82×10^6^ nm^3^), as gained from the 3D model, also shows an increase in comparison to WT (2.25±0.80×10^6^ nm^3^; Figure 1H). However, when analyzing 10 complete nuclear volumes in *mal3Δ* cells by ultra thin serial section electron microscopy, we found that only seven nuclear envelopes had a clear SPB associated with them. Two further nuclei had electron dense material that could not be unambiguously recognized as an SPB and one final nucleus had no SPB at all (Figure S1 in File S1). Yet, using the SPB marker Cut12-GFP (see below)[29], we found a clear fluorescent dot in each cell, indicating the presence of an SPB.

To see if deletion of *tip1* would also have effects on the SPB morphology; seven SPBs of *tip1Δ* cells in log-phase culture were examined using serial sections. The *tip1Δ* SPBs appeared comparable to *mal3Δ* SPBs with larger width and total volume than WT SPBs (2.98±1.05×10^6^ nm^3^ in *tip1Δ*; Figure 1E–H).

### Increased SPB cdc11-GFP and cut12-GFP Signal in *tip1Δ* and *mal3Δ* Mutants

To confirm the EM results we decided to correlate the fluorescence intensity of two GFP labeled SPB proteins with cell length in *mal3Δ* and *tip1Δ* mutants. We decided to use the *S. pombe* centriolin homologue Cdc11p. This essential protein localizes to the SPB, and is necessary for septum formation [30]. In addition, we used Cut12p, a protein necessary for activation and integration of the SPB into the nuclear envelope during mitosis [31].

Cdc11-GFP and Cut12-GFP in WT cells showed unchanged fluorescence until cells reached ∼13 µm, where cells usually enter mitosis (Figure S2 in File S1). In both *mal3Δ* and *tip1Δ* mutants the fluorescent signal from Cut11-GFP and Cut12-GFP was increased throughout the cell cycle (Figure 1K and Figure S2 in File S1, n = 90, 92, and 95 for Cut12-GFP in WT, *mal3Δ*, and *tip1Δ* resp., n = 126, 113, and 97 for Cdc11-GFP in WT, *mal3Δ*, and *tip1Δ* resp.). The average SPB signal was roughly 150% of that measured for the corresponding WT cells, in line with the tomographic data. Thus, this increased fluorescence intensity in *tip1Δ* and *mal3Δ* mutants supports our EM data revealing structural SPB alterations.

One could imagine that this increase in SPB fluorescence is an indirect effect of both mutants having short MTs. Therefore, we measured the fluorescence intensity of SPBs in cells treated with MBC (a MT poison), which leaves short MT ‘stubs’ [32], and compared them with untreated cells. The Cut12-GFP and Cdc11-GFP signals in MBC treated cells did not change compared to untreated cells (Figure 1K, Figure S2 in File S1, n = 333 and 280 for Cut12-GFP with and without MBC resp., n = 286 and 404 for Cdc11-GFP with and without MBC resp.). This suggests that the increased fluorescence seen in the +TIP mutants *mal3Δ* and *tip1Δ* is specific, uncovering possible novel functions for these proteins in maintaining SPB morphology.

### Changes in MT - MTOC Interaction in *mal3Δ* and *tip1Δ* Mutants

**Figure 2:**
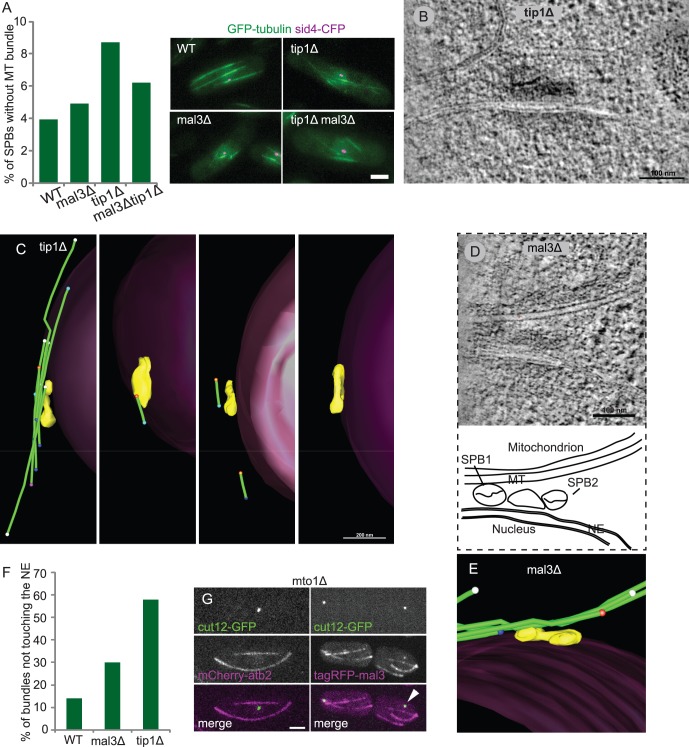
Mal3p and Tip1p are both involved in MT bundle anchoring to the NE/SPB. **A)** Fluorescence microscopy of cells expressing Sid4-CFP and GFP-tubulin show a disruption in SPB-MT interaction in mutant cells. Scale bar 2 µm **B)** Slices from a tomogram reveal relatively normal SPB morphology in *tip1Δ* mutants. **C)** 3D models of MT bundle association with the SPB in four different *tip1Δ* cells. **D)** Tomographic slice showing a duplicated SPB in a *mal3Δ* mutant. SPB1 is in close association with a MT, whereas the MT bends away from SPB2. **E)** A 3D model of the entire SPB of which a slice was shown in D). Note that no MTs are associated with the right SPB. **F)** A large percentage of 3D models of bundles in *tip1Δ* and *mal3Δ* mutants were not associated with the NE. **G)** Green channel red channel, and merged image of *mto1Δ* cells expressing Cut12-GFP and either mCherry-Atb2 (left panels) or Mal3-tagRFP (right panels). The centre cell of the left panel shows an SPB without attached MTs. The two cells of the right panels have SPBs without MTs but a punctate Mal3p signal co-localizes with both SPBs. Scale bar 2 µm.

We wondered if these changes in SPB morphology in *mal3Δ* and *tip1Δ* mutants altered the SPB function as an MTOC. In WT cells, MT bundles are attached to specialized interphase MTOCs (iMTOCs) located around the nuclear envelope and to the SPB (see [28], [33]). We examined the SPB/MT bundle relationship using fluorescence microscopy of cells expressing GFP-tubulin and the SPB marker Sid4-CFP [34]. Sid4p is a stable SPB protein that acts as an anchor for components of the septation initiation network like Cdc11p [35]. In interphase, 4% of WT cells had no MTs co-localizing with Sid4-CFP (n = 202). In the +TIP mutants this number was 5% in *mal3Δ* and 9% in *tip1Δ* mutants (n = 203 and 138 cells respectively). In *tip1Δ* and *mal3Δ* double mutant cells, 7% of cells had an SPB with no associated MTs (n = 225; Figure 2A).

In *tip1Δ* cells, four SPBs were reconstructed using electron tomography. The SPB morphology appeared normal (Figure 2B), with clear electron densities and an electron dense plaque, but only two SPBs had attached MTs. The third SPB had one MT at approximately 25 nm distance and the final SPB had no associated MTs (Figure 2C). Additionally, two duplicated SPBs in *mal3Δ* cells of rather normal morphology were studied by electron tomography (Figure 2D). In both cases MTs were only in contact with one of the SPBs, indicating that the other SPB was functionally immature (Figure 2E; Movie S2). These results underscore the fact that the high spatial resolution of EM can yield results which would not be found using traditional fluorescence microscopy.

To examine the MT/nuclear envelope interaction we quantified the percentage of MT bundles detached from (i.e. not in direct contact with) the nuclear envelope. In tomograms of WT cells, 14% of single MTs or bundled MTs (n = 14 bundles) were nuclear envelope-disassociated. In tomograms of *tip1Δ* and *mal3Δ* cells, MTs were generally found close to the nucleus but we noticed an increased detachment from the NE. 58% of MTs or MT bundles (n = 19 bundles) that we studied in *tip1Δ* cells were away from the NE (Figure 2F). In *mal3Δ,* 30% of the single MTs or bundled MTs were not in contact with the NE (n = 27 bundles).

Therefore, Mal3p and Tip1p each have a direct effect on MT attachments to SPBs and iMTOCs, giving these +TIP proteins an important role in the spatial organization of MTs.

### Does Mal3p Localize to SPBs Independent of MTs?

In order to regulate MT attachment to the SPBs we might expect Mal3p to localize to SPBs in addition to its reported localization to the MT lattice and the MT minus ends *in vitro* [2], [7]. We attempted but failed to detect Mal3p at the SPB by using an on-section immunogold approach with anti-Mal3p or anti-GFP antibodies (Figure S1 in File S1). This could be due to technical problems such the inaccessibility of the SPB for antibodies or a low number of Mal3p proteins at the SPB. In an alternative approach, we used an *mto1Δ* mutant in which interphase MT nucleation is abolished. The observed interphase MTs originate as spindle MTs which escape the nucleus after mitosis [36], [37]. These MTs are frequently detached from the SPB and nucleus, and allowed us to study SPB constituents in the absence of MTs. Comparing *mto1Δ* cells expressing mCherry-tubulin with cells expressing Mal3-tagRFP in addition to Cut12-GFP (to label the SPBs), we found Mal3p signal associated with 34% (n = 71) of SPBs without associated MT-bundles. In contrast 94% (n = 102) of bundle-free SPBs showed no tubulin signal (Figure 2G). This result indicates that Mal3p could potentially be a structural component of the SPB.

### MT Organization in *tip1Δ* and *mal3Δ* Cells

We have previously shown that the MT bundle associated with the SPB is stabilized relative to other interphase MT bundles [28]. Do the disturbed MT/SPB and MT/iMTOCs interactions and the altered SPB morphology change the general MT bundle organization?

Detailed organization of the architecture of MT bundles cannot be visualized by light microscopy, due to its limits in resolution. Therefore, we applied large-scale electron tomography to high-pressure frozen, freeze substituted interphase *tip1Δ* and *mal3Δ* cells (5 and 8 cells respectively).

To ensure that only cells in early G2 were examined, we selected for the shortest cells we could find (7.2±0.51 µm *mal3Δ* and 7±0.4 µm *tip1Δ*). This is longer than the WT cells we studied previously [28], which suggests that mutants divide at a greater length than WT cells. This was confirmed when cell length was measured in septating WT and mutant cells using light microscopy (WT 12.9±1.5 µm n = 100 cells, *mal3Δ* 16.4±1.9 µm n = 97 and *tip1Δ* 16.5±1.6 µm n = 87; Figure S3 in File S1). Thus, the mutant cells examined here are likely to be at a cell cycle stage that is comparable to those previously imaged [28].

**Figure 3:**
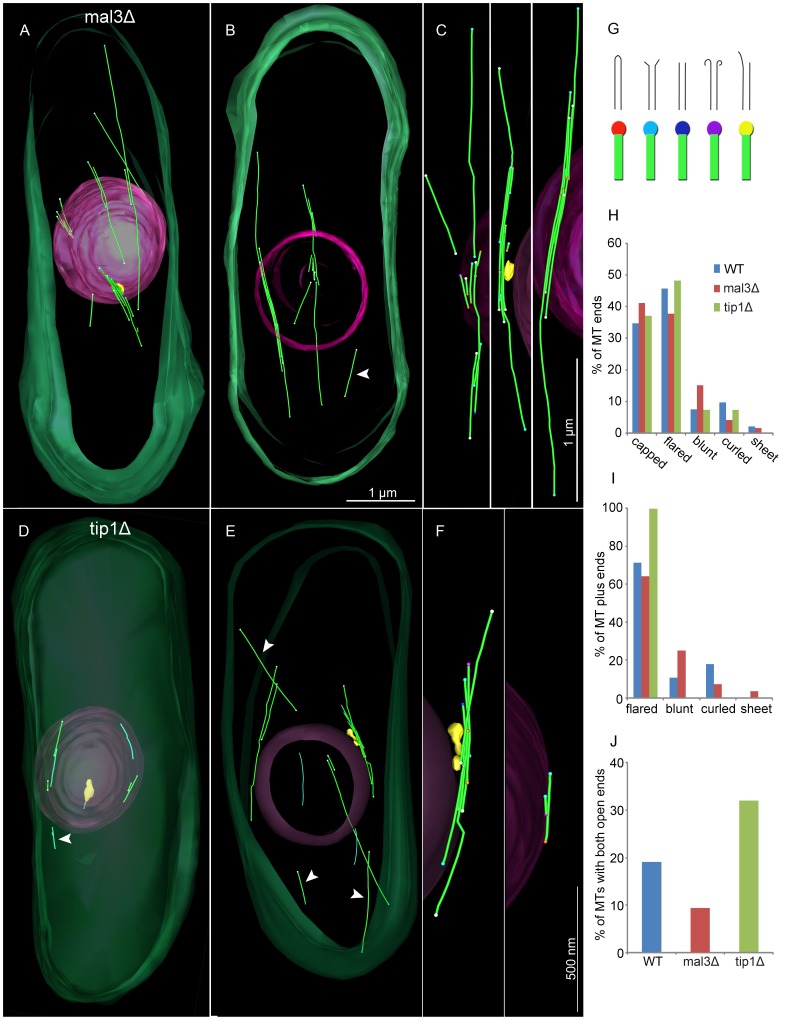
MT bundle organization in *mal3Δ* is similar to WT but disturbed in *tip1Δ*. Using serial section electron tomograms 3D model of cells and individual MT bundles was made. **A–C)** 3D model of *mal3Δ* cells, and **D–F)** *tip1Δ* cells. In the model, plasma membrane is colored transparent green, the nuclear envelope transparent pink, SPBs in yellow, MTs in green and in their ends colored balls that represent their end structure. **G)** A line drawing of the different MT ends structures found and their color code in the model. **H)** Distribution of MT end structures, both on minus and plus ends. I) The distribution of end structures at MT plus ends. **J)** MTs with both open ends were found in all three cell strains, but they were more common in cells lacking Tip1p.

In 3D models of *mal3Δ* mutants, the MT bundles showed no major organizational differences compared to WT cells, though the MTs in mutant cells were identifiably shorter (Figure 3A–C; Movie S3). The individual MTs within the bundle were on average 0.65±0.55 µm long (n = 55) corresponding to ∼40% of the average WT MT length. Bundles contained 4.8±4.1 MTs compared to 4.4±2.6 MTs in WT cells (Figure S3 in File S1).

In *tip1Δ* cells, on the other hand, bundles generally contained less than half the number of MTs (1.7±1.6 MTs/bundle) compared to WT cells (Figure 3D–F). Additionally, individual MTs were short, 0.68±0.57 µm (n = 33; Figure S3 in File S1).

We reconstructed one full cell volume of a *tip1Δ* cell, enabling comparison with the previously published complete WT cell volume (Figure 3D; Movie S4, [28]). Instead of the 16 MT filaments found in the WT cell, the *tip1Δ* cell contained only 10 MTs. Together, these 10 filaments comprised 3.8 µm of polymerized tubulin, a 89% reduction compared to the length found in the WT cell (34.5 µm). Furthermore, the amount of incorporated tubulin may be even further decreased due to the structural changes described next. We conclude that MT filament nucleation or maintenance appears affected in *tip1Δ*, but not in *mal3Δ* mutants.

### MT End Morphology in +TIP Mutants

Since +TIPs localize specifically to plus ends of MTs where they influence their dynamic properties, a change in MT plus end morphology could occur in cells lacking such proteins. Using electron tomography, we examined individual MT ends and grouped them according to their structure as capped, flared, blunt, curled and sheeted (Figure 3G; Table S1 in File S1; [28], [32]).

**Figure 4:**
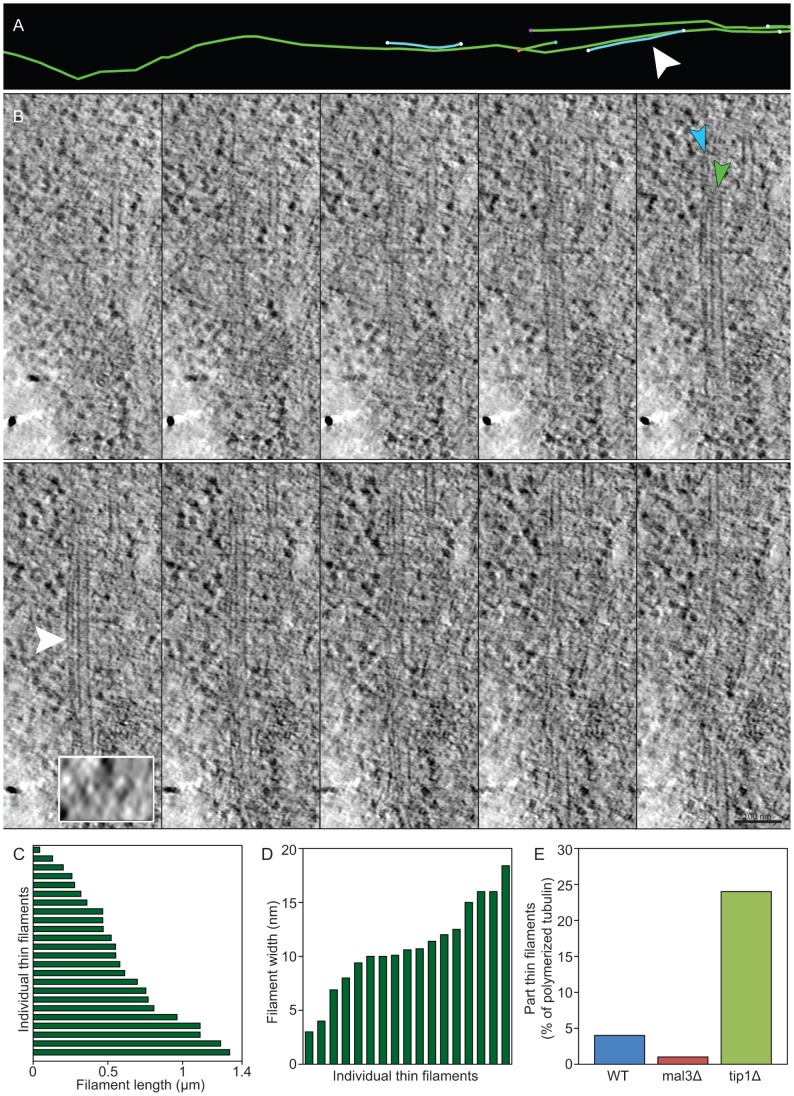
tip1Δ has an increased proportion of thin filaments. **A)** A wild type MT bundle (green), containing two thin filaments (turquoise). The white arrowhead points to the filament shown in B. **B)** Slices from the tomogram (every 3 nm) showing a thin hollow filament next to a normal MT. The insert is a snapshot of the marked position (turquoise arrowhead) rotated 90 degrees in the x-axis, so that the filaments are visible in cross-section. This clearly shows that both filaments are hollow and that they have different diameters. **C–D)** The lengths and widths of individual thin filaments **E)** The proportion of the total polymerized tubulin incorporated into thin filaments in WT, *mal3Δ* and *tip1Δ* clearly shows an increase in these filaments in *tip1Δ.*

As in WT cells, flared ends are the most commonly found MT end structure in *mal3Δ* and *tip1Δ* mutants (WT 46% n = 92 ends, *mal3Δ* 38% n = 119 ends, and *tip1Δ* 50% n = 26 ends; Figure 4H), closely followed by capped ends (35%, 41% and 35%, respectively). Blunt ends were the third most common structure, with increased prevalence in *mal3Δ* cells (15% of ends in comparison to 7% and 8% in WT and *tip1Δ*). Curled ends were slightly more common in WT (10%) in comparison to 4% and 7% in *mal3Δ* and *tip1Δ* mutants. Ends displaying curved sheets were the most uncommon structure of all with only 2% of ends in WT and *mal3Δ*, and sheets were never seen in *tip1Δ.*

Since the minus ends of yeast cell MTs are normally capped [38], the opposed end is the MT plus end [32]. Additionally, if a MT had two ends with the same end structure, one of them must be a plus end and the other a minus end. Using these criteria to identify MT plus ends, we found 71% (WT n = 28 MTs), 64% (*mal3Δ* n = 28) and 100% (*tip1Δ* n = 7) of the MT plus ends to be flared (Figure 3I). Blunt ends were more common in *mal3Δ* mutants (25% versus 11% in WT).

In all three strains, MTs with two open ends were found (WT 19%, *mal3Δ* 9% and *tip1Δ* 32%), showing that not all MT nucleation occurs at a γ-TURC or that this association gets lost after nucleation (Figure 3J).

### Structural Changes to the MT Lattice of *tip1Δ* and *mal3Δ* Mutants

+TIPs are known to modify MT dynamics *in vivo*, but little is known in which manner this regulation occurs. We asked whether these +TIP proteins are important for MT lattice integrity. In all *S. pombe* cells reconstructed, we have seen thin, hollow filaments aligned with MT bundles (Figure 4A–B; Movie S5). These filaments are 0.04 to 1.3 µm long (0.60±0.35 µm; n = 24 from all three strains; Figure 4C). They are often hollow tubes for a part of the length and then change to be just a filament or a couple of filaments. This makes their diameter variable. However, when measured on the widest point, we found filaments with diameters ranging from ∼3 nm (single filament, not tube) to ∼18 nm (the diameter of most MTs in our sample). The average thin filament diameter was 10.8±4.1 nm (n = 17; Figure 4D). Taken together with their staining properties, and their total absence in cells treated with MBC (n = 94; Figure 4E), lead us to interpret the filaments as an alternate assembly form of tubulin. In WT and *mal3Δ* cells, these filaments encompassed 4% and 1% respectively of the total filament lengths in the reconstructed cells (Figure 4E). In contrast, 24% of all filaments in *tip1Δ* cells were displaying this thin character. Thus, the lack of Tip1p in the deletion strain generates abnormally thin filaments, likely indicating an important function of Tip1p in stabilizing evenly calibrated MT polymers. Alternatively, Tip1p may play a role in normal nucleation of MTs. The higher proportion of thin filaments could also be an indirect effect of the large decrease of ‘normal’ MTs in *tip1Δ* whilst these thin structures are left unaffected.

**Figure 5:**
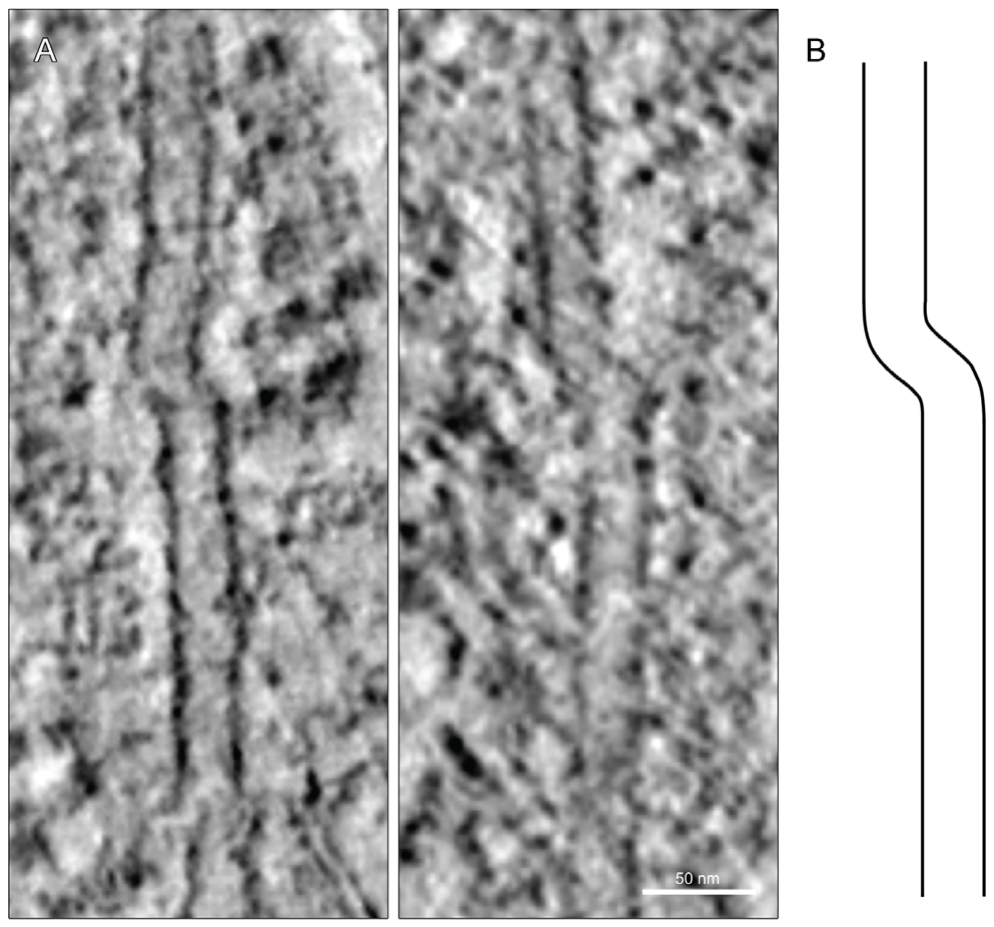
Microtubules lacking Mal3p have ‘kinks’. **A)** Interphase microtubules with ‘kinked’ lattice, which were never observed in WT. **B)** line drawing of a kinked microtubule.

We studied a total of 120 MTs in *mal3Δ* cells, which sums up to 78 µm polymer length. Four of these MTs contained a ‘kink’ in the lattice (Figure 5A–B). Hence, we had one kink per 19.5 µm polymer. In WT cells no such kinks were found, when studying a total polymer length of 121 µm spread over 87 filaments. We also observed adjacent MT ends facing each other as if they had been the same MT but broken in two parts in *mal3Δ* cells (Movie S6). Thus, we have found two different lattice defects in the two +TIP mutants examined.

## Discussion

We have shown that the absence of either one of the two +TIP proteins, Tip1p or Mal3p, alters the morphology of the SPB, and the anchoring of MTs to the SPB (and iMTOCs). We hypothesize that this could result from three different causes; 1) both +TIP mutants have short MTs which could affect the SPB morphology by decreased pushing forces. 2) The short MTs are probing a smaller area of the cytoplasm and thus encounter different proteins, or different concentrations of proteins that are delivered to the SPB. 3) These +TIPs are true structural SPB components.

The two first hypotheses both depend on short MTs. Previous studies have shown that using MBC slightly increases SPB size in *S. cerevisiae* [39], which would agree with our findings. However the increase in Cut12p and Cdc11p signal seen in *tip1Δ* and *mal3Δ* mutants was absent in MBC treated cells. Thus, the SPB phenotypes seen here are probably not merely an indirect effect of short MTs.

The third hypothesis, that these +TIPs may be structural SPB components, is in agreement with the fact that Mal3p and Tip1p homologues (i.e. proteins of the EB1 and CLIP-170 families) have been found close to SPBs and centrosomes in a variety of organisms using light microscopy [14]–[19].

For instance, EB1 localization to the centrosomes was shown to be unaffected during drug-induced MT depolymerisation [14], [16]. However, our recent paper [32] shows that short stubs of MTs might remain after depolymerisation using a drug.

Thus, the EB1 localization described by light microscopy, both in untreated and drug treated cells, might be a consequence of EB1 localizing to plus or minus ends of short MTs close to MTOCs.

Furthermore, we could not localize Mal3p to the SPB ultrastructure using anti-Mal3p antibodies nor anti-GFP antibodies on thin sections (Figure S2 in File S1).

However, EB1 localizes to isolated centrosomes, devoid of MTs, from both *Dictyostelium* and mammalian cells [14], [19]. The importance of the EB1 C-terminus for localization to the centrosome, whereas the MT binding domain is found on its N-terminus, also points toward a specific binding at this location [19].

EB1 has been shown to anchor MTs to the area around the centrosome [15], [19], [26], a result which is reinforced by our data showing that only one SPB is in contact with MTs in *mal3Δ* cells containing duplicated SPBs and a general dysfunction in MT attachment to SPBs and iMTOCs in both +TIP mutants. Both EB1 and CLIP-170 homologues have been shown to bind to centrosomal proteins [24], [26]. We also show evidence that Mal3p localizes to SPBs without attached MT-bundles. These results point towards a notion that Mal3p and perhaps also Tip1p may be structural SPB components.

There is some controversy as to when the SPB matures in *S. pombe* cells. Ding et al. [27] reported that using serial thin section EM, only single SPBs were found in logarithmically growing WT cells examined during early G2. In serial sections of cells arrested in the cell cycle using starvation or genetic mutations, SPBs were found to be duplicated at the G1/S phase transition [40]. It should be noted, that in the latter study, any ambiguous SPBs were scored as duplicated, and cells released from arrest underwent multiple successive cell divisions.

We have used logarithmically growing cells, which were synchronized using elutriation, a physical method based on centrifugation. Short cells were harvested and left to recover for one cell cycle before they were cryoimmobilized for electron microscopy. Modeling of serial section reconstructions showed that both single and duplicated SPBs were present in cells with G2 lengths, although duplicated SPBs were twice as common. The presence of single SPBs in these cells may indicate that duplication is not occurring exclusively at the G1/S transition.

Fluorescence microscopy shows Mal3-GFP localization to growing MT plus ends as well as faint staining along MT lattices. Further, *in vitro* data has shown that Mal3p localizes to the seam of the MT lattice [7]. Therefore, deletion of *mal3* could have an effect on the MT lattice *in situ*. We suggest that the kinked MTs seen could be an effect of the weakened seam. Large numbers of MTs in this kinked state would not be found since the strain in the lattice would destabilize the structure. The increase in thin MTs in *tip1Δ* mutants is another example of the unexpected influence of a +TIP on MT structure.

Understanding +TIP action at the minus end is challenging using light microscopy because of the proximity of MT ends and MTOCs. We have shown with a combination of light and electron microscopy/tomography that Tip1p and Mal3p have important roles in MTOC function, such as MT anchoring to the SPB and iMTOCs. Indeed, their absence even causes structural alterations to the SPB and MTs. +TIP function at the minus ends/MTOCs is a novel field, in which these proteins will likely have different interaction partners and patterns than at the rather well studied MT +end [1], [41]. Therefore, it will be essential to understand the full impact of the +TIPs on the centrosome/SPB and MT minus ends in order to appreciate the full extent to which these proteins are involved in MT, MTOC, and global cellular regulation.

## Materials and Methods

### Live Imaging and Image Analysis

Logarithmically growing cells in minimal medium (EMM2), with appropriate amino acid supplements (with or without thiamine) were attached to glass bottom dishes (MatTek, Ashland, MA) using 1–2 µl of lectin (2 mg/ml, Sigma, St. Louis, MO). Unattached cells were washed away after 10 minutes and attached cells were imaged using a Coolsnap HQ camera (Roper Scientific, Tucson, AZ) on an Axiovert 200 M microscope (Zeiss, Göttingen, Germany; Plan-Apo100× NA 1.4 objective) on a PerkinElmer Ultraview RS spinning disc confocal microscope (PerkinElmer, Waltham, USA) 488 nm and 565 nm laser lines; with an Apo 100×/1.30 NA oil objective, or an Andor revolution spinning disk confocal microscope (Andor Technology plc., Belfast, Northern Ireland, UK) using 488 nm and 565 nm laser lines; with an Apo 100×/1.40 NA oil objective. Cells were grown and imaged at 25°C. All strains used are listed in Table 1. Image analysis was carried out using Image J software and the EMBL plugin collection. Further image processing was carried out with adobe photoshop.

**Table 1:** Strains used in this study.

Strain	Genotype	Origin
**DB427**	h- tip1Δ:: kanR leu1-32 ura4-D18 ade6-M210	[3]
**DB483**	h- tip1Δ:: kanR	[3]
**DB558**	h-	[44]
**DB713**	h- mal3Δ::his3+ his3-D1 ura4-D18	[3]
**DB1219**	h- cut12:GFP::ura4+ leu1-32 ura4-D18	[29]
**DB1237**	h- cut12:GFP:ura4+ mal3Δ::his3+ ura4-D18 leu1-32 his3-D1 ade6-M210	This study
**DB1326**	h- cdc11:GFP::kanR ura4-D18 ade6-M210 leu1-32	[30]
**DB1376**	h- cdc11:GFP::kanR mal3Δ::his3+ his3-D1	This study
**DB1599**	h- tip1Δ::kanR cut12:GFP::ura4+ leu-1-32 ura4-D18	This study
**DB1870**	h- cdc11:GFP::kanR tip1Δ:: kanR ura4-D18 ade6-M210 leu1-32	This study
**DB2855**	h? sid4:CFP:: kanR lys1+::nmt1p:GFP:atb2 mal3Δ::his3+ ade6-M210 leu1-32 ura4-D18 his3-D1	This study
**DB2856**	h? sid4:CFP:: kanR lys1+::nmt1p:GFP:atb2 mal3Δ::his3+ ade6-M210 leu1-32 ura4-D18 his3-D1	This study
**DB2857**	h? sid4:CFP:: kanR lys1+::nmt1p:GFP:atb2 tip1Δ:: kanR mal3Δ::his3+ ade6-M210 leu1-32 ura4-D18 his3-D1	This study
**DB2864**	h? sid4:CFP:: kanR lys1+::nmt1p:GFP:atb2 leu1-32 ura4-D18	This study
**DB3169**	h? mto1Δ:: kanR cut12:GFP::ura4+ aur1+::mCherry:atb2 ade6-m21(0/6) leu1-32 ura4-D18	This study
**DB3257**	h? mto1Δ::kanR mal3:HL:tagRFP::kanR cut12:GFP::ura4+ ura4-D18 leu1-32 ade6-m216	This study

### SPB Intensity Measurements

Fluorescence intensity of the SPB in WT cells expressing either Cut12-GFP (DB1219) or Cdc11-GFP (DB1326), was compared with *mal3Δ cut12:GFP* (DB1237) or *mal3Δ cdc11:GFP* (DB1376) and *tip1Δ cut12:GFP* (DB1599) or *cdc11:GFP* (DB1870) cells. The cells were imaged on the Zeiss microscope. To create maximum Z-projections, Image J was used on Z-stacks with images acquired every 0.5 µm. To measure fluorescence intensity, the total fluorescence in a fixed sized region of interest (ROI) around the SPB was extracted, and the background fluorescence subtracted using Image J.

Cells expressing either Cut12-GFP (DB1219) or Cdc11-GFP (DB1326) were treated with 25 µg/ml MBC or DMSO (control) for 50 minutes prior to imaging on the PerkinElmer microscope. Images were analysed using a custom-made Matlab (Mathworks) programme. Briefly, the images were corrected for background (both darkfield and fluorescence) and the SPBs were identified as bright dots using the canny algorithm. Fluorescent intensities of the five slices around the centre of each SPB were taken to calculate the total fluorescent intensity of each SPB. Results were plotted using SigmaPlot (Systat Software, Inc., Chicago, USA). To compare the results of the experiments with different microscopes, treatments, and different tagged proteins, we normalized our results. Briefly, in all conditions the fluorescence intensity of the tagged SPB proteins in the WT control was set to 100%. The fluorescence intensity measured in the mutants/MBC treated cells was adjusted with the same factor to allow comparison with WT controls.

### SPB Attachment to MT Bundles

Wild type (DB1116), *tip1Δ* (DB1864), and *mal3Δ* (DB1867) cells, all expressing GFP-Atb2 and Sad1-dsRed, were imaged on the Zeiss microscope. Cells showing any Sad1-dsRed signals unattached to a MT-bundle were counted as having an unattached SPB. More than 100 cells/strain were analysed.

### tagRFP-Mal3 and mCherry:atb2 at Bundle Free SPBs

Mutants for *mto1* expressing Cut12-GFP and either mCherry-Atb2 (DB3169) or tagRFP-Mal3 (DB3173) were imaged on the Andor microscope. Cells that had no SPB attached MT-bundle but did show a punctate Atb2p or Mal3p signal at the SPB were counted. More than 100 cells/strain were analysed.

### Sample Preparation for Electron Microscopy

Logarithmically growing *mal3Δ* (DB518) and *tip1Δ* (DB427) cells (in rich, YE5S, medium) were harvested through filtration and high pressure frozen followed by freeze substitution and embedding into HM20 as in [28], [42]. For the biometric analysis of SPBs, WT (DB558) and *mal3Δ* (DB713) cells had been synchronized using centrifugal elutriation, and high pressure frozen after 140 minutes of recovery time. Cells were then in early G2, as controlled by light microscopy. Mutants for *tip1* (DB427; in log-phase growth) were filtered and frozen. The cells were then embedded into Epon 812 (Carl Roth, GmbH, Karlsruhe, Germany).

### 3D Reconstruction of Nuclei and SPBs

To reconstruct whole nuclei and SPBs, we used serial thin sections (80 nm) to include the whole object of interest, including an extra section on each side to ensure no material was missed. In these sections, nuclei or SPBs in short cells (early G2; Figure S3A) were digitally imaged using a KeenView 1 K wide screen CCD camera (1376×1032 pixels) (Soft Imaging System, Muenster, Germany) on a Biotwin CM120 (FEI, Eindhoven, The Netherlands), operated at 100 kV. The images of serial sections were then aligned to each other in MIDAS [43]. The nuclei were analyzed and the presence/absence of a readily identifiable SPB was scored.

To analyze SPB size in synchronized early G2 cells, contours were drawn around the SPBs using the IMOD software suite [43], producing 3D models representative of the nucleus/SPBs. The width and lengths of SPBs were measured on these 3D models. The volumes of SPBs were extracted using the IMODINFO program. Their duplication status was analyzed visually.

### Electron Tomography

For electron tomography, cells were sectioned into 250 nm thick slices and then imaged at 14500×magnification in 1–1.25° increments over a ±65° range using a Gatan Ultrascan 890 or 895 (pixel size 1.5 nm; Pleasanton, CA, USA) on a FEI Tecnai TF20 electron microscope (Hillsboro, Oregon, USA). Montaged single-axis tomograms of serial sections were reconstructed, joined and 3D models of features of interest were generated using the IMOD software [42]. MT lengths were extracted using IMODINFO. MT ends were examined in all available directions and categorized as in [28], [32]. The SPB model in Figure 2B–D was made using Amira software (Visage Imaging GmbH, Berlin, Germany).

## Supporting Information

Figure S1 (related to Figure 1): SPB maturation and morphology in WT and +TIP mutants. **A)** Serial thin sections of a WT SPB in a cell undergoing cytokinesis, showing no signs of duplication. **B)** The cell in which the SPB was found is clearly undergoing cytokinesis (past G1/S phase). **C)** Serial thin sections of a whole nucleus without recognizable SPB in a *mal3Δ* cell. **D–E)** Immunocytochemistry on thin sections failed to localize Mal3p and Tip1p to the SPB. Cells were prepared by high pressure freezing and freeze substitution in 0.1% UA and 1% H_2_O in acetone for 50 hours and then embedded into HM20. **D)** The strain/antibody combinations we used. All secondary antibodies were protein A gold conjugated. **E)** Two unlabeled WT SPBs. The absence of labeling could be caused by 1) too few epitopes, since these have to be displayed on the surface on the section. Hence, our trial with Mal3-GFP over expression but this also failed to localize gold to the SPB. 2) The epitopes were altered during sample preparation. 3) Mal3p and Tip1p might not be localized to the SPB.
(PDF)

Figure S2 (related to Figure 1): Increased SPB Cdc11-GFP and Cut12-GFP signal in *tip1Δ* and *mal3Δ* mutants. **A)** Both GFP tagged SPB proteins, Cut12p and Cdc11p, show increased fluorescence intensity in the +TIP deletion mutants. (Cut12-GFP: WT 23±12 a.u n = 90, *mal3Δ* 36±14 a.u. n = 92, *tip1Δ* 43±20 a.u n = 95. Cdc11-GFP: WT 31±22 a.u. n = 126, *mal3Δ* 49±32 a.u. n = 113, *tip1Δ* 40±35 a.u. n = 97) **B)** WT and MBC treated cells show no difference in SPB fluorescence intensity, showing that the short MTs are not enough to change the SPBs (11837±4255 a.u. n = 331 versus 12213±4803 a.u. n = 280 in untreated vs treated cells). ± indicates SD, n = number of cells.
(PDF)

Figure S3 (related to Figure 3): G2 Cells are longer and microtubules shorter in both +TIP mutants. A) Phase contrast images of septating cells show the difference in length between WT and the +TIP mutants at the time of division. The box plot shows the distribution of cell lengths at septum formation. Average cell length for septating WT was 12.9±1.5 µm (n = 100 cells), *mal3Δ* 16.4±1.9 µm (n = 97 cells) and *tip1Δ* 16.5±1.6 µm (n = 87 cells). B) Statistics on MT lengths and number of MTs in a bundle from all the cells examined. Only MTs both starting and ending in the reconstructed volume were integrated in this analysis. C–D) Each graph displays the individual MT lengths found in the reconstruction from one (partial) cell. The SPB bundle is marked with a star. E) The MT length distributions found in all the cells of the two +TIP mutants.
(PDF)

Table S1 Numbers of MTs displaying each combination of end structures.
(PDF)

File S1 **Table S1 and Figures S1, S2, and S3.**
(PDF)

Movie S1 **3D SPB morphology in a WT cell.** This movie steps through 1 nm thick tomographic slices of a duplicated SPB that sits on the nuclear envelope. On the third pass, the 3D model of the SPB is added, and then visualized on its own revealing the 3D architecture of this amorphous electron density in transparent gold, the central bridge in yellow and the central plaques in red. The SPB is 270 nm long.
(MOV)

Movie S2 **Only one SPB is touching the MTs in** ***mal3Δ*** **mutants.** This movie steps through 1 nm thick tomographic slices of a duplicated SPB that sits on the nuclear envelope. On the second pass, the 3D model of the SPB (yellow) and associated MTs (green) sitting on the nuclear envelope (pink) is added, and then visualized on its own showing how the MTs never touch the second of the SPBs. From the morphology we cannot say which SPB is the mother and which one is the daughter SPB. Flared MT ends are shown in turquoise, capped ends in red, blunt ends in blue and ambiguous ends in white.
(MOV)

Movie S3 **MT 3D architecture in a partial** ***mal3Δ*** **cell reconstruction show shorter MT filaments arranged in a WT manner.** 3D model made from 9 serial section montaged tomograms. The plasma membrane has been modeled in transparent dark green, the nuclear envelope in pink, SPB in yellow, MTs in green, flared ends in turquoise, ambiguous ends in white, capped ends in red and curled ends in purple. Scale bar is 500 nm.
(MOV)

Movie S4 **A whole cell reconstruction of** ***tip1Δ*** **mutants reveals few and short MTs.** 3D model made from 17 serial section montaged tomograms. The plasma membrane has been modeled in transparent dark green, the nuclear envelope in pink, SPB in yellow, MTs in green, flared ends in turquoise, ambiguous ends in white, capped ends in red, and curled ends in purple. Scale bar is 500 nm.
(MOV)

Movie S5 **Thin filaments were found in all cell types, but more commonly in** ***tip1Δ*** **mutants.** A series of 1.5 nm thick tomographic slices shows two filaments, one is a clear MT and the parallel filament is only about half its thickness. Scale bar 50 nm.
(MOV)

Movie S6 **Closely opposed open ends indicate MT breakage in** ***mal3Δ*** **cells.** A series of 1.5 nm thick tomographic slices shows two flared MT ends (black arrows) within 40 nm of each other. The movie first images through the raw data, and then returns through the same region with the painted model added for clarity. MT has been modeled in green and flared ends in turquoise. Scale bar is 20 nm.
(MOV)
