# Supplementary material for: Electron Tomography Reveals Novel Microtubule Lattice and Microtubule Organizing Centre Defects in +TIP Mutants
Source: PLoS One. 2013 Apr 16;8(4):e61698. doi: 10.1371/journal.pone.0061698 (PMC3627915; doi:10.1371/journal.pone.0061698)

Figure S1

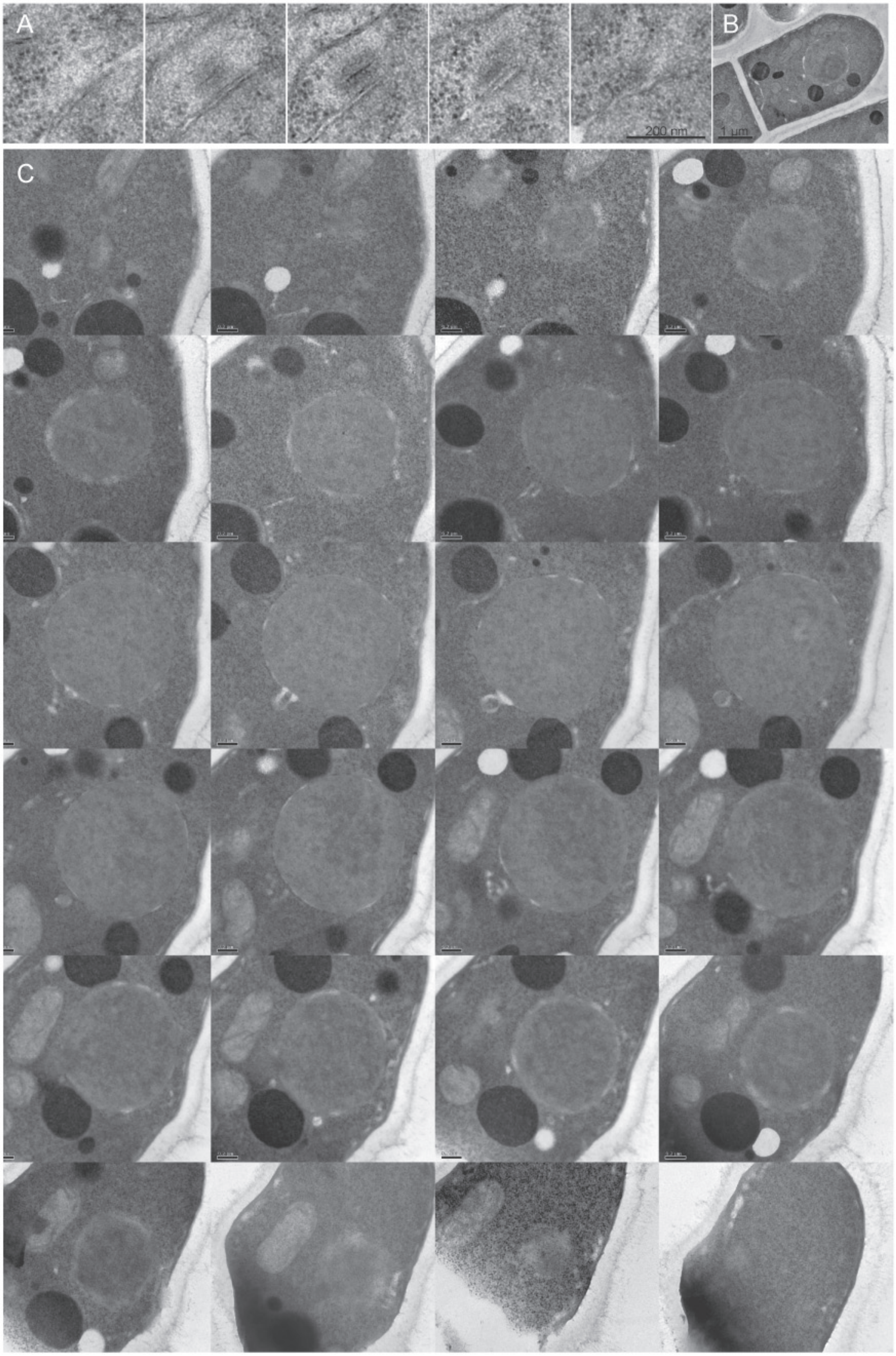


D

| Strain | 1° antibody |
|---|---|
| GFP-Mal3 | Living colours rabbit anti-GFP antibody (Clontech, CA, USA) or goat anti-GFP antibody (Rockland Immunochemicals Inc., PA, USA) |
| Tip1-GFP | Same as above |
| GFP-Mal3 over expression | Same as above |
| WT | Rabbit polyclonal anti-Mal3p, 3 different mouse anti-Mal3p mono-clonal antibodies, a mixture of said 3 monoclonal antibodies |

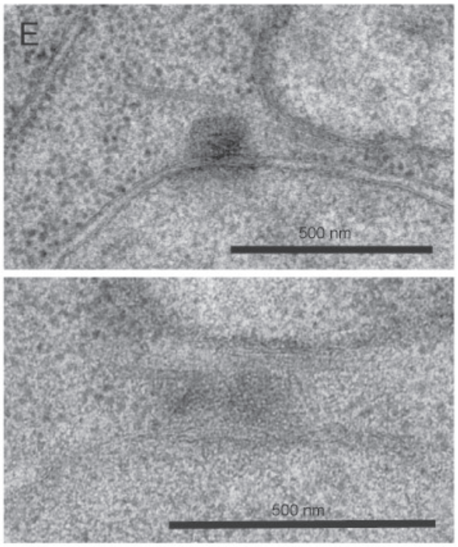

Supplement: Figure S1 — (related to Figure 1): SPB maturation and morphology in WT and +TIP mutants. A) Serial thin sections of a WT SPB in a cell undergoing cytokinesis, showing no signs of duplication. B) The cell in which the SPB was found is clearly undergoing cytokinesis (past G1/S phase). C) Serial thin sections of a whole nucleus without recognizable SPB in a mal3Δ cell. D–E) Immunocytochemistry on thin sections failed to localize Mal3p and Tip1p to the SPB. Cells were prepared by high pressure freezing and freeze substitution in 0.1% UA and 1% H2O in acetone for 50 hours and then embedded into HM20. D) The strain/antibody combinations we used. All secondary antibodies were protein A gold conjugated. E) Two unlabeled WT SPBs. The absence of labeling could be caused by 1) too few epitopes, since these have to be displayed on the surface on the section. Hence, our trial with Mal3-GFP over expression but this also failed to localize gold to the SPB. 2) The epitopes were altered during sample preparation. 3) Mal3p and Tip1p might not be localized to the SPB. (PDF) [file pone.0061698.s001.pdf]
