# Supplementary figures and images for: Electron Tomography Reveals Novel Microtubule Lattice and Microtubule Organizing Centre Defects in +TIP Mutants
Source: PLoS One. 2013 Apr 16;8(4):e61698. doi: 10.1371/journal.pone.0061698 (PMC3627915; doi:10.1371/journal.pone.0061698)

Figure S2

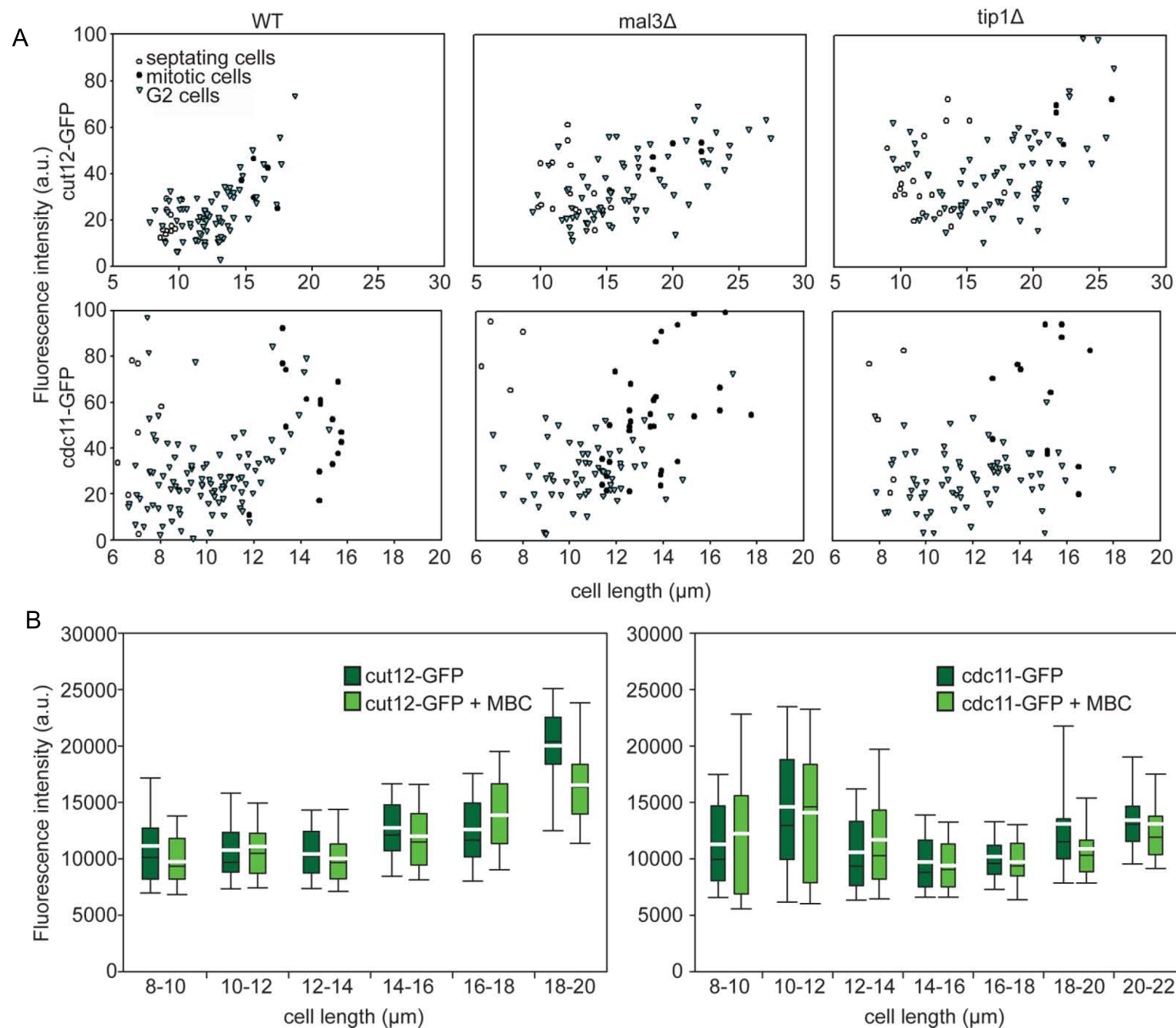

Supplement: Figure S2 — (related to Figure 1): Increased SPB Cdc11-GFP and Cut12-GFP signal in tip1Δ and mal3Δ mutants. A) Both GFP tagged SPB proteins, Cut12p and Cdc11p, show increased fluorescence intensity in the +TIP deletion mutants. (Cut12-GFP: WT 23±12 a.u n = 90, mal3Δ 36±14 a.u. n = 92, tip1Δ 43±20 a.u n = 95. Cdc11-GFP: WT 31±22 a.u. n = 126, mal3Δ 49±32 a.u. n = 113, tip1Δ 40±35 a.u. n = 97) B) WT and MBC treated cells show no difference in SPB fluorescence intensity, showing that the short MTs are not enough to change the SPBs (11837±4255 a.u. n = 331 versus 12213±4803 a.u. n = 280 in untreated vs treated cells). ± indicates SD, n = number of cells. (PDF) [file pone.0061698.s002.pdf]

Figure S3

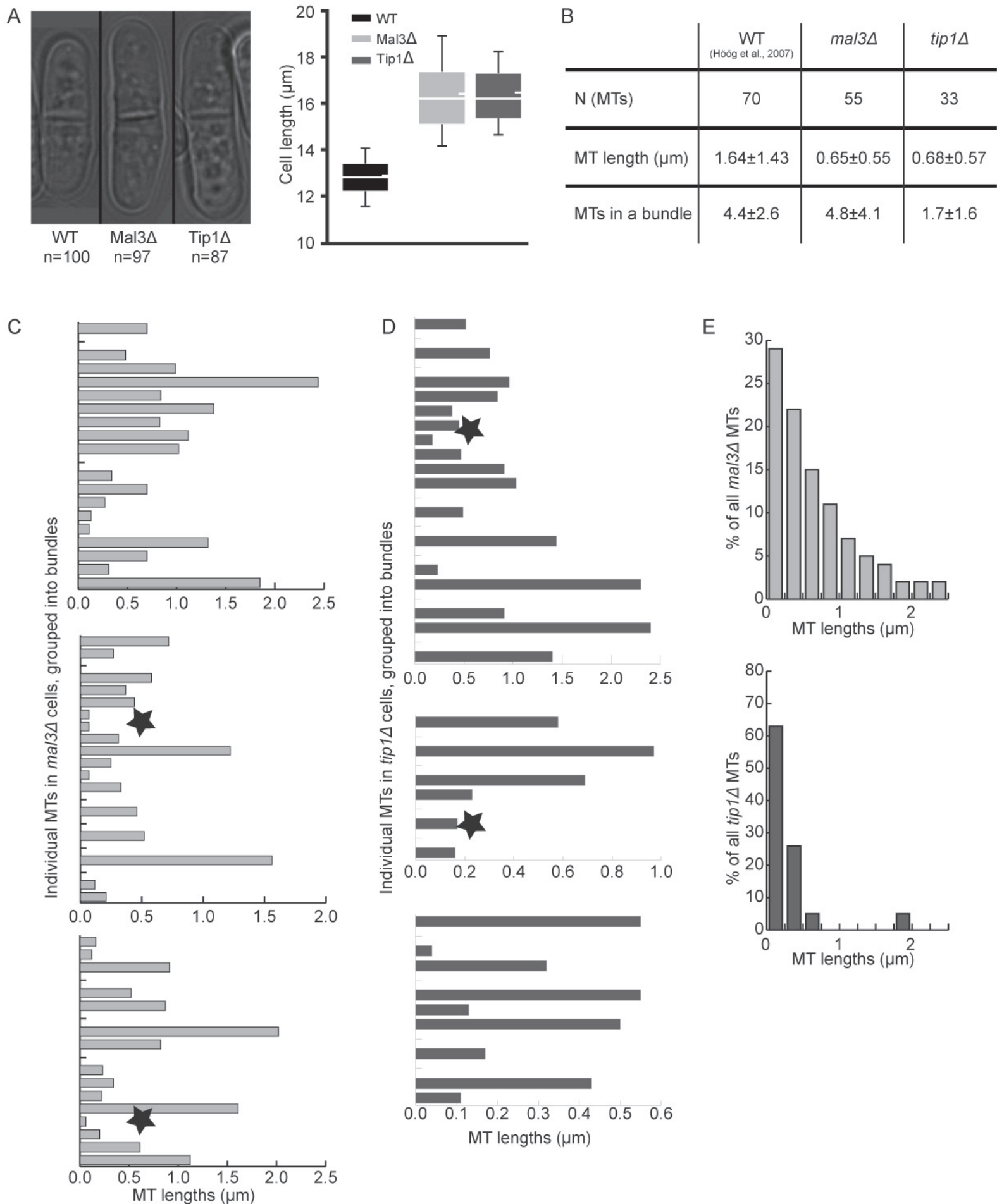

Supplement: Figure S3 — (related to Figure 3): G2 Cells are longer and microtubules shorter in both +TIP mutants. A) Phase contrast images of septating cells show the difference in length between WT and the +TIP mutants at the time of division. The box plot shows the distribution of cell lengths at septum formation. Average cell length for septating WT was 12.9±1.5 µm (n = 100 cells), mal3Δ 16.4±1.9 µm (n = 97 cells) and tip1Δ 16.5±1.6 µm (n = 87 cells). B) Statistics on MT lengths and number of MTs in a bundle from all the cells examined. Only MTs both starting and ending in the reconstructed volume were integrated in this analysis. C–D) Each graph displays the individual MT lengths found in the reconstruction from one (partial) cell. The SPB bundle is marked with a star. E) The MT length distributions found in all the cells of the two +TIP mutants. (PDF) [file pone.0061698.s003.pdf]
