# Supplementary material for: Electron Tomography Reveals Novel Microtubule Lattice and Microtubule Organizing Centre Defects in +TIP Mutants
Source: PLoS One. 2013 Apr 16;8(4):e61698. doi: 10.1371/journal.pone.0061698 (PMC3627915; doi:10.1371/journal.pone.0061698)
Supplement: Table S1 — Numbers of MTs displaying each combination of end structures. (PDF) [file pone.0061698.s004.pdf]

# Table S1

**Table S1: Numbers of MTs displaying each combination of end structures. Amb. = ambiguous, representing both unclassifiable ends, and ends not found within the reconstructed volume**

| | capped/flared | capped/curled | capped/blunt | capped/sheet | flared/flared | blunt/blunt |
|---|---|---|---|---|---|---|
| **WT** | 15 | 5 | 3 | 0 | 5 | 0 |
| ***Mal3Δ*** | 17 | 2 | 6 | 1 | 2 | 1 |
| ***Tip1Δ*** | 6 | 0 | 0 | 0 | 1 | 0 |

| | sheet/sheet | curled/curled | flared/blunt | flared/sheet | flared/curled | blunt/sheet |
|---|---|---|---|---|---|---|
| **WT** | 0 | 1 | 3 | 2 | 2 | 0 |
| ***Mal3Δ*** | 0 | 0 | 5 | 0 | 1 | 0 |
| ***Tip1Δ*** | 0 | 0 | 0 | 0 | 1 | 0 |

| | blunt/curled | curled/sheet | flared/amb. | amb./amb. | capped/amb. | curled/amb. |
|---|---|---|---|---|---|---|
| **WT** | 0 | 0 | 10 | 7 | 9 | 0 |
| ***Mal3Δ*** | 0 | 0 | 18 | 12 | 23 | 2 |
| ***Tip1Δ*** | 0 | 0 | 4 | 7 | 3 | 1 |

| | blunt/ambiguous | sheet/ambiguous |
|---|---|---|
| **WT** | 1 | 0 |
| ***Mal3Δ*** | 5 | 1 |
| ***Tip1Δ*** | 2 | 0 |
