# Supplementary material for: Electron Tomography Reveals Novel Microtubule Lattice and Microtubule Organizing Centre Defects in +TIP Mutants
Source: PLoS One. 2013 Apr 16;8(4):e61698. doi: 10.1371/journal.pone.0061698 (PMC3627915; doi:10.1371/journal.pone.0061698)
Supplement: File S1 — Table S1 and Figures S1, S2, and S3. (PDF) [file pone.0061698.s005.pdf]

Figure S1

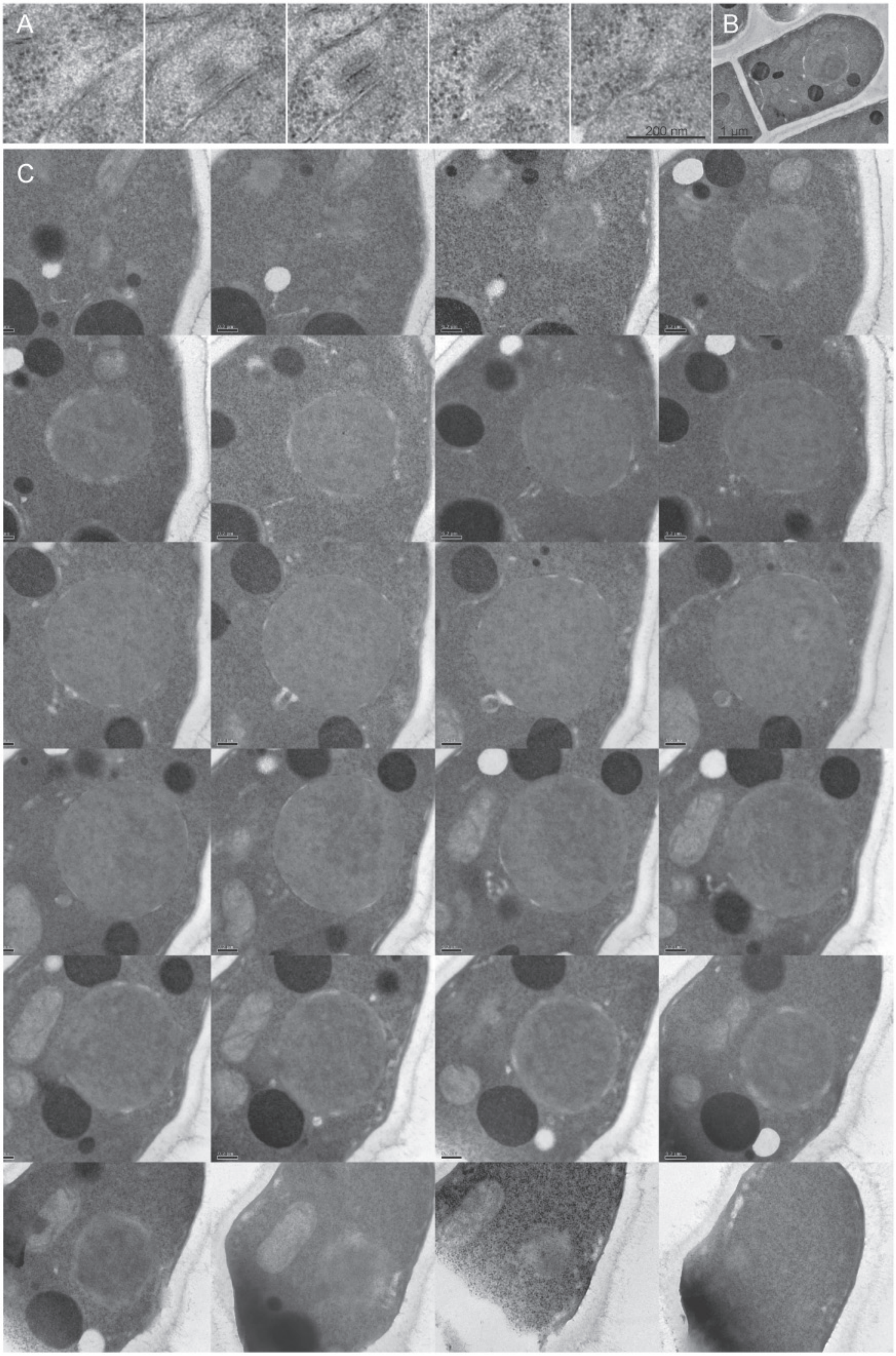

D Strain	1° antibody
GFP-Mal3	Living colours rabbit anti-GFP antibody (Clontech, CA, USA) or goat anti-GFP antibody (Rockland Immunochemicals Inc., PA, USA)
Tip1-GFP	Same as above
GFP-Mal3 over expression	Same as above
WT	Rabbit polyclonal anti-Mal3p, 3 different mouse anti-Mal3p mono-clonal antibodies, a mixture of said 3 monoclonal antibodies

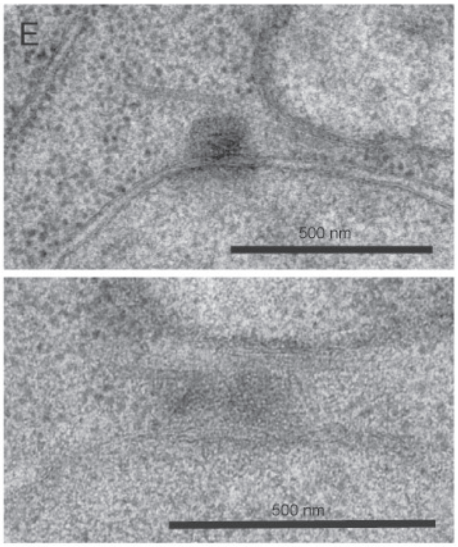

Figure S2

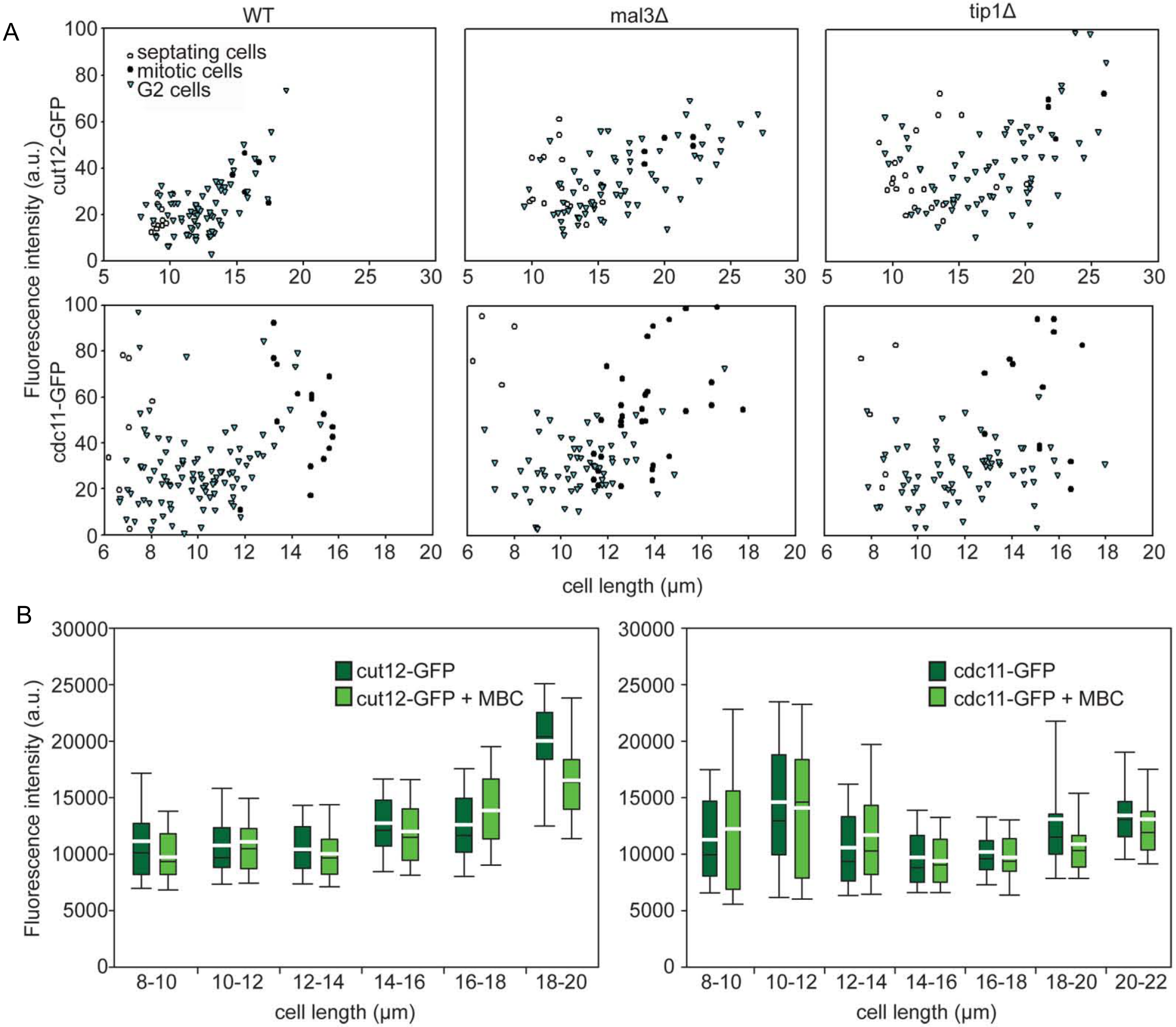
A
WT
mal3Δ
tip1Δ
septating cells
mitotic cells
G2 cells
Fluorescence intensity (a.u.)
cut12-GFP
cdc11-GFP
cell length (µm)
B
cut12-GFP
cut12-GFP + MBC
cdc11-GFP
cdc11-GFP + MBC
Fluorescence intensity (a.u.)
8-10
10-12
12-14
14-16
16-18
18-20
20-22
cell length (µm)

Figure S3

A

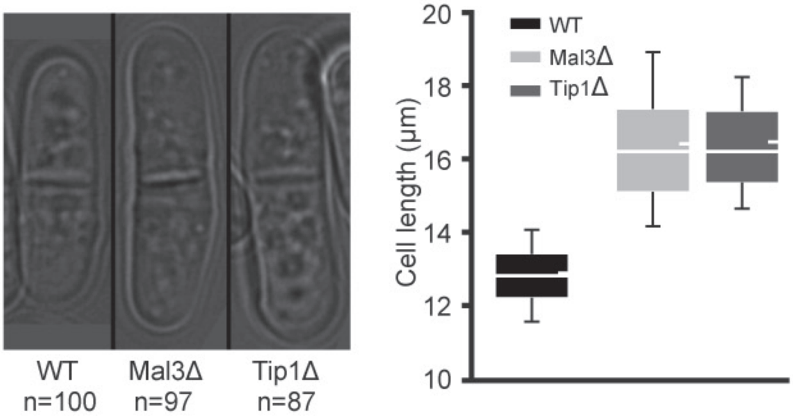

B

	WT (Höög et al., 2007)	*mal3Δ*	*tip1Δ*
N (MTs)	70	55	33
MT length (μm)	1.64±1.43	0.65±0.55	0.68±0.57
MTs in a bundle	4.4±2.6	4.8±4.1	1.7±1.6

C D E

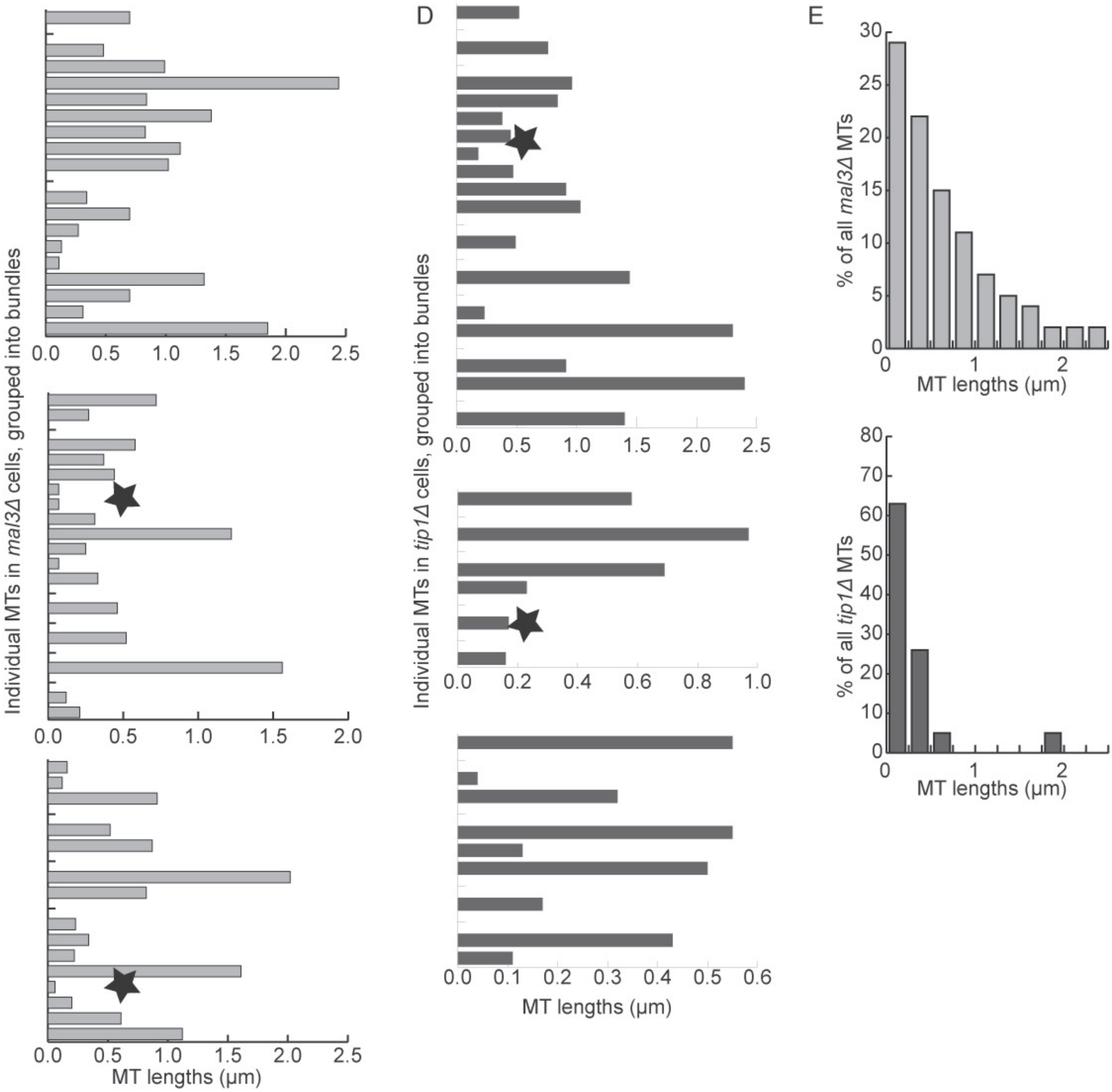

Table S1

Table S1: Numbers of MTs displaying each combination of end structures. Amb. = ambiguous, representing both unclassifiable ends, and ends not found within the reconstructed volume

	capped/flared	capped/curled	capped/blunt	capped/sheet	flared/flared	blunt/blunt
**WT**	15	5	3	0	5	0
Mal3Δ	17	2	6	1	2	1
Tip1Δ	6	0	0	0	1	0

	sheet/sheet	curled/curled	flared/blunt	flared/sheet	flared/curled	blunt/sheet
**WT**	0	1	3	2	2	0
Mal3Δ	0	0	5	0	1	0
Tip1Δ	0	0	0	0	1	0

	blunt/curled	curled/sheet	flared/amb.	amb./amb.	capped/amb.	curled/amb.
**WT**	0	0	10	7	9	0
Mal3Δ	0	0	18	12	23	2
Tip1Δ	0	0	4	7	3	1

	blunt/ambiguous	sheet/ambiguous
**WT**	1	0
Mal3Δ	5	1
Tip1Δ	2	0
